# Charge-transfer crystallites as molecular electrical dopants

**DOI:** 10.1038/ncomms9560

**Published:** 2015-10-06

**Authors:** Henry Méndez, Georg Heimel, Stefanie Winkler, Johannes Frisch, Andreas Opitz, Katrein Sauer, Berthold Wegner, Martin Oehzelt, Christian Röthel, Steffen Duhm, Daniel Többens, Norbert Koch, Ingo Salzmann

**Affiliations:** 1 Humboldt-Universität zu Berlin, Institut für Physik and IRIS Adlershof, AG Supramolekulare Systeme, Brook-Taylor Straße 6, 12489 Berlin, Germany; 2 Departamento de Física, Pontificia Universidad Javeriana, Carrera 7, No. 43–82 Ed. 52 Of. 606, Bogotá, Colombia; 3 Helmholtz-Zentrum Berlin für Materialien und Energie GmbH, Bereich Solarenergieforschung, Albert-Einstein-Straße 15, 12489 Berlin, Germany; 4 Institut für Festkörperphysik, Graz University of Technology, Petersgasse 16, Graz 8010, Austria; 5 Jiangsu Key Laboratory for Carbon Based Functional Materials and Devices and Institute of Functional Nano and Soft Materials (FUNSOM), Soochow University, 199 Ren-Ai Road, Suzhou 215123, China; 6 Soochow University-Western University Joint Centre for Synchrotron Radiation Research (SWC) and Collaborative Innovation Center of Suzhou Nano Science & Technology (NANO-CIC), Soochow University, 199 Ren-Ai Road, Suzhou 215123, China; 7 Helmholtz-Zentrum Berlin für Materialien und Energie GmbH—BESSY II, Abteilung Kristallographie, Albert-Einstein-Straße 15, 12489 Berlin, Germany

## Abstract

Ground-state integer charge transfer is commonly regarded as the basic mechanism of molecular electrical doping in both, conjugated polymers and oligomers. Here, we demonstrate that fundamentally different processes can occur in the two types of organic semiconductors instead. Using complementary experimental techniques supported by theory, we contrast a polythiophene, where molecular *p*-doping leads to integer charge transfer reportedly localized to one quaterthiophene backbone segment, to the quaterthiophene oligomer itself. Despite a comparable relative increase in conductivity, we observe only partial charge transfer for the latter. In contrast to the parent polymer, pronounced intermolecular frontier-orbital hybridization of oligomer and dopant in 1:1 mixed-stack co-crystallites leads to the emergence of empty electronic states within the energy gap of the surrounding quaterthiophene matrix. It is their Fermi–Dirac occupation that yields mobile charge carriers and, therefore, the co-crystallites—rather than individual acceptor molecules—should be regarded as the dopants in such systems.

## Abstract (web summary)

The fundamental mechanisms of doping organic semiconductors are poorly understood compared with their inorganic counterparts. Here, the authors demonstrate that small conjugated molecules and conjugated polymers exhibit fundamentally different phenomena upon doping despite similar compositions.

## Graphical abstract

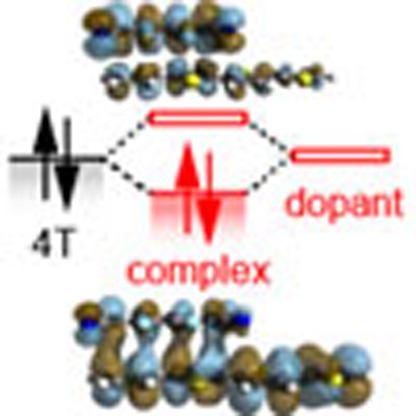

Doping inorganic semiconductors by introducing impurity atoms is the basis of all functionality in modern electronic devices. It allows deliberately tuning the band alignment at interfaces and markedly increases the conductivity at ultralow doping ratios, as typically every covalently bound dopant atom donates one mobile charge to the highly crystalline and ultra-pure semiconductor matrix^1^. However, their costly production makes inorganic semiconductors less attractive for large-area applications and, for opto-electronic applications in particular, the tuning of their bandgap still remains a technological challenge. In these fields, organic electronics emerged as a valuable alternative, where organic semiconductors (OSCs), that is, conjugated organic molecules (COMs) and conjugated polymers (CPs), are employed as active materials instead. In addition to the organic light-emitting devices revolutionizing today's display technology, OSCs show great potential also for future applications in organic photovoltaic cells, field-effect transistors and sensors^2^,^3^,^4^,^5^. Clearly, however, a similar variety of applications, as it is feasible with inorganic semiconductors, can only be realized with OSCs if their electrical doping can be similarly well controlled.

Despite intense research since the early days of organic electronics^2^, doping OSCs by introducing alkali metals or halides proved to be inappropriate for device applications due to their tendency to diffuse^6^,^7^. As a valuable alternative, molecular electrical doping has emerged^8^,^9^,^10^,^11^,^12^,^13^,^14^, which employs strong molecular acceptors for *p*-type doping and donors for *n*-type doping instead. Exploiting the full wealth of organic chemistry, molecular dopants are designed such that, for *p*-type doping, their electron affinity (EA) is in the range of the ionization energy (IE) of the OSC, and vice versa for *n*-type doping. In today's benchmark devices, this approach is ubiquitously pursued, in particular for conduction layers in organic light-emitting devices and organic photovoltaic cells (*p–i–n* devices)^4^,^10^ as well as in organic field-effect transistors^11^.

Given its success in practical applications, it may come as a surprise that the fundamental processes at work in the molecular doping of OSCs are still little understood. In one common perception, ground-state integer charge transfer (ICT) is thought to occur, leading to a localized charge on the ionized dopant and a (mobile) hole in the OSC matrix, for example, a positive polaron for *p*-type doping^10^,^15^. For CPs, a mounting body of experimental evidence, much of it gathered on the benchmark polymer poly(3-hexylthiophene) shown as P3HT in Fig. 1a, indeed points towards the validity of this view: (i) a marked increase in conductivity by up to five orders of magnitude (up to ∼1 S cm^−1^) was reported upon molecularly *p*-doping P3HT with the strong electron acceptor 2,3,5,6-tetrafluoro-7,7,8,8-tetracyanoquinodimethane (F4TCNQ)^16^,^17^,^18^. (ii) In Fourier-transform infrared (FTIR) spectroscopy, characteristic shifts of F4TCNQ cyano-vibrations were observed that indicate its fully ionized state^19^,^20^. (iii) In ultraviolet/visible/near-infrared (UV/Vis/NIR) absorption spectroscopy, optical transitions characteristic for the fully ionized species were observed for the F4TCNQ doping of P3HT^16^,^17^,^21^. (iv) New electronic states were found via ultraviolet photoelectron spectroscopy (UPS) at the Fermi energy (*E*_F_) upon doping P3HT with NOPF_6_ (ref. 22), as it was expected for positive polarons according to the model for polaron energetics of Su, Schrieffer and Heeger^23^,^24^.

However, only a small fraction of the transferred integer charges were found to give rise to mobile holes for conduction with the rest of the hole/dopant–anion pairs remaining Coulombically bound^25^. Further investigations on copolymers comprising thiophene units of different length led to the notion that the ICT process itself is, in fact, localized to only one quaterthiophene (4T) unit of the polymer, with neighbouring pairs of dopant and 4T units not interacting notably^20^. This correlates with the results of Duong *et al*.^16^, who reported a maximum in conductivity for around that ratio. Also Gao *et al*.^26^ found the response in electron paramagnetic resonance spectroscopy, proportional to the density of unpaired spins, to steadily increase up to a saturation ratio of one F4TCNQ molecule per 4T unit in P3HT. Together, these findings seem to suggest that, upon molecular doping with F4TCNQ, quaterthiophene should exhibit very much the same phenomenology as the parent polymer P3HT^18^,^20^,^26^.

In the present work, we explore whether and, if so, to which extent 4T can indeed be regarded as a model system for understanding the doping of P3HT. By combining a number of complementary experimental techniques with theoretical modelling on the density functional theory (DFT) and semi-classical level, we reveal striking differences in the phenomenology of molecularly doped thiophene oligo- and polymers, which allows concluding on inherent differences in the fundamental processes at work for the two material classes. While the doping-induced relative increase in conductivity seems comparable between P3HT and 4T films, the ICT scenario found for P3HT does not occur for *p*-doped 4T, despite a similar microstructure. Instead, our study suggests the exclusive formation of ground-state charge-transfer complexes with pronounced intermolecular frontier-orbital hybridization in 1:1 mixed-stack co-crystals of dopant and host, which precipitate from the surrounding OSC matrix and take over the role of the dopant in the oligomer system.

## Results

### Sheet conductivity

**Figure 1:**
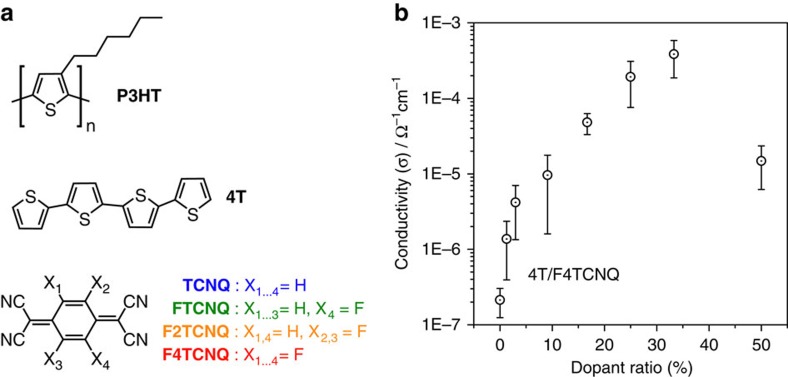
Materials and lateral conductivity of doped 4T films. (**a**) Chemical structures of the materials employed: P3HT and 4T as OSCs and the increasingly fluorinated TCNQ derivatives FTCNQ, F2TCNQ and F4TCNQ employed as *p*-dopants. (**b**) Lateral thin-film conductivity as a function of the dopant ratio (that is, the number of dopant molecules divided by total number of molecules) for vacuum co-deposited films of 4T and F4TCNQ; for information on the error margin see the Methods section.

In analogy to P3HT^16^,^17^, admixing F4TCNQ to 4T effectively increases its lateral thin-film conductivity (*σ*), as shown in Fig. 1b. Initially, *σ* shows a steep increase by about one order of magnitude for a doping concentration as low as 1.3%, which corresponds to one dopant per 75 host molecules. Higher dopant loading steadily increases *σ* to a maximum value more than three orders of magnitude higher than that of the pristine 4T film. In line with common observations in the molecular doping of all types of OSCs, *σ* eventually saturates^16^,^27^,^28^,^29^,^30^. In contrast to P3HT, however, saturation is reached already around one *p*-dopant per two quaterthiophene units and conductivity is significantly reduced in 1:1 blends of dopant and host (50% dopant ratio), where the maximum was observed for the polymer^16^.

### Vibrational spectroscopy

**Figure 2:**
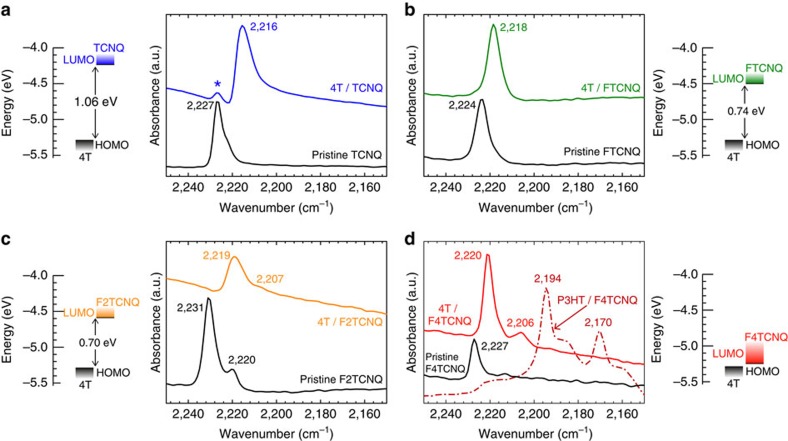
Diagnostic cyano-vibrations and energy levels. FTIR spectra in the characteristic cyano-stretching region for 4T films in 1:1 blends (50% dopant ratio) with the acceptors (**a**) TCNQ, (**b**) FTCNQ, (**c**) F2TCNQ and (**d**) F4TCNQ; pristine 4T does not exhibit any vibrational bands in the region displayed. HOMO denotes the highest occupied molecular orbital of 4T, LUMO the lowest unoccupied molecular orbital of the respective dopant species in the energy level diagrams; the IE of 4T was determined by UPS (*cf*. Fig. 4b), EA values of the dopants are taken from literature^9^,^33^. The minor peak marked by an asterisk in **a** is assigned to excess and, therefore, charge-neutral TCNQ; in **d**, the spectrum for an F4TCNQ-doped P3HT film (28.6% dopant ratio, that is, one dopant per 2.5 quaterthiophene segments of the polymer backbone) is shown as reference.

To more thoroughly investigate the process underlying the increase of *σ* on a molecular scale, we carried out FTIR on 1:1 mixed films of 4T and F4TCNQ. Shifts of characteristic cyano-vibrational bands in FTIR are held to indicate the negatively charged state of F4TCNQ^19^,^31^ and to allow quantifying the degree of charge transfer (*δ*). In these studies, *δ* is reported to linearly scale with the observed frequency shift (Δ*ν*) following


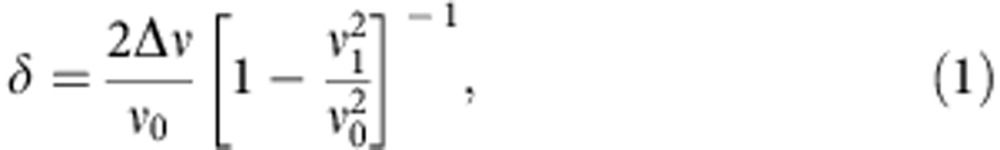


where *ν*_0_ and *ν*_1_ denote the vibrational frequencies of the neutral dopant and that of its radical anion, respectively^32^. To assess also the impact of the dopant strength (that is, its EA) on *δ*, we additionally employed the weaker dopants TCNQ, FTCNQ and F2TCNQ (see chemical structures in Fig. 1a) covering, therewith, a range in EA of almost 1 eV (determined by inverse photoemission)^33^, as indicated in the energy level diagram insets in Fig. 2, where all FTIR results are summarized.

It is known that, for alkali salts of the respective dopants, *δ* equals essentially 1 and the frequency of the strongest nitrile-stretching mode shifts from the neutral value of *ν*_0_=2,227 cm^−1^ to *ν*_1_=2,183 cm^−1^ for TCNQ (Δ*ν*=44 cm^−1^) and *ν*_1_=2,194 cm^−1^ for F4TCNQ (Δ*ν*=33 cm^−1^)^31^,^34^. Importantly, for F4TCNQ, a shift of identical magnitude is observed upon doping P3HT (Fig. 2d), hence indicating ICT for the *p*-doped polymer^20^. In marked contrast, however, for the present 4T blends with TCNQ as the weakest acceptor, only a small shift of Δ*ν*=11 cm^−1^ is found, which translates into a partial charge transfer of *δ*=0.25 via equation (1). Strikingly, for F4TCNQ as a significantly stronger dopant with an EA higher by ca. 1 eV through perfluorination^9^, Δ*ν* is now even lower (Δ*ν*=7 cm^−1^), corresponding to a charge transfer of only *δ*=0.21; this value is in excellent agreement with DFT calculations yielding *δ*=0.24. Likewise, for all intermediate EA cases, comparable Δ*ν* values of 6 cm^−1^ (FTCNQ) and 12 cm^−1^ (F2TCNQ) are experimentally observed. All the shifted vibrational bands are notably well defined and of comparable peak width, which indicates essentially identical *δ* for all the individual dopant molecules throughout the samples. Importantly, the bands are also not significantly broadened compared with the neutral film, which points towards a well-defined environment of the dopant molecules in both 4T and P3HT.

### Optical spectroscopy

**Figure 3:**
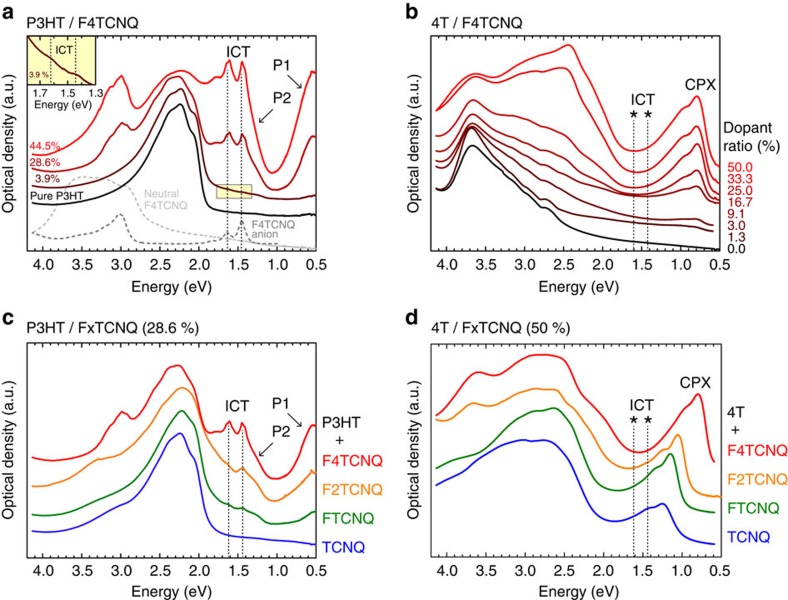
Optical absorption spectra of molecularly doped poly- and quaterthiophene. UV/Vis/NIR spectra of (**a**) P3HT blended with F4TCNQ at increasing ratio with the inset showing the zoomed region around the ICT features in the film of 3.9% dopant ratio (one F4TCNQ per 25 quaterthiophene segments), (**b**) 4T blended with F4TCNQ at increasing ratio, (**c**) P3HT blended with the full range of differently strong dopants (TCNQ to F4TCNQ) at a dopant ratio of 28.6% and (**d**) of 4T blends with the full range of differently strong dopants (TCNQ to F4TCNQ) in 1:1 ratio (50%); P1 and P2 indicate the optical transitions of the positive polaron in P3HT^21^,^35^,^36^,^37^; asterisks indicate the expected transition energies related to dopant anions (ICT) that are absent in the 4T case.

Recent studies deduced ICT as the fundamental mechanism in the F4TCNQ doping of P3HT from the occurrence of new sub-bandgap absorptions in UV/Vis/NIR spectroscopy^16^,^17^,^25^. As can be seen in Fig. 3a, sharp features characteristic for the dopant radical anion are observed already at the low dopant ratio of 4% (that is, one dopant per 25 quaterthiophene segments), which then increase in intensity with the dopant loading. In addition, broad absorptions are seen to arise, which are interpreted as the two allowed optical transitions, P1 and P2, of the positive polaron in P3HT^21^,^25^,^35^,^36^,^37^. Together with *δ*=1 as deduced from FTIR^20^, this evidences ICT in P3HT, not only when *p*-doped with F4TCNQ, but also for the weaker dopants F2TCNQ and FTCNQ. For the weakest in the series, TCNQ, no sub-bandgap features are observed, as ICT is no longer expected with its EA=4.23 eV (ref. 33) (Fig. 2a) lower than the polymer IE=4.60 eV (ref. 38). To compare these data with the present case of the *p*-doped 4T oligomer with its low *δ*-values indicating well-defined, but only partial charge transfer instead, we also performed UV/Vis/NIR; the results are depicted in Fig. 3b together with reference data of pristine 4T. In contrast to P3HT, no indications of ionized F4TCNQ molecules are observed here whatsoever (expected F4TCNQ anion transitions are marked with stars). Instead, fundamentally different sub-gap absorptions arise already at the lowest doping ratio of 1.3%, which equals one dopant molecule per 75 4T-oligomers. These features, assigned to a single electronic transition with vibronic replica on the basis of our time-dependent DFT results (Supplementary Fig. 1), and increase in intensity with dopant loading while retaining their transition energy up to the case of 1:1 blends (50% dopant ratio, that is, one dopant per 4T molecule). Further employing the full range of differently strong dopants yields analogous results, notably also for TCNQ, with no spectroscopic indication for ionized species (Fig. 3b). However, a clear trend for the energy of the new sub-gap transition is apparent, which is lower in energy for dopants of higher EA. This observation further allows unambiguously excluding these transitions to stem from 4T radical cations^39^,^40^, because the related polaronic transition energies are not expected to depend on the EA of the dopant species. Note that these findings are independent of the preparation method, as fully analogous results are found for solution-processed 4T films employing the same experimental protocol used for P3HT (Supplementary Fig. 2 and Supplementary Table 1). As the IE of 4T (5.3 eV, *vide infra*) is still slightly higher than the EA of F4TCNQ (maximum literature value: 5.24 eV)^9^, we further employed the even stronger, chemically slightly different dopant 2,2′-(perfluoro-naphthalene-2,6-diylidene)dimalononitrile (F6TCNNQ)^29^,^41^ with an EA as high as 5.6±0.2 eV, which, however, led to qualitatively identical results (Supplementary Figs 3–5). Overall, above spectroscopic findings for the thiophene oligomer are in striking contrast to the ICT phenomenology observed for P3HT and beg for a different explanation.

### Photoelectron spectroscopy

**Figure 4:**
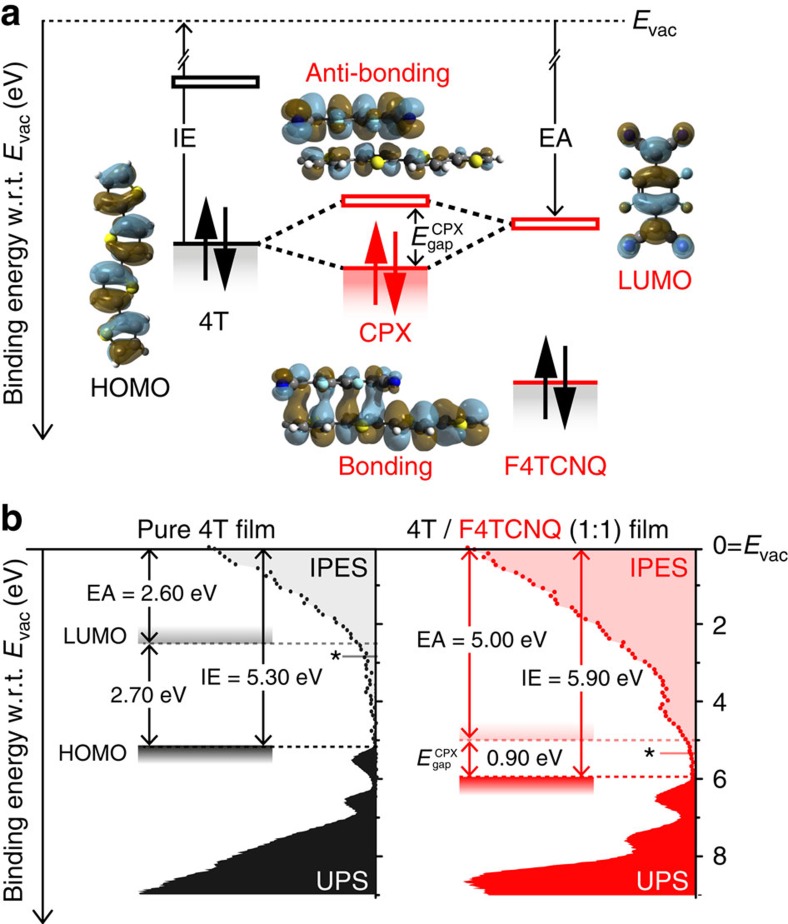
Energies of supramolecular hybrid orbitals in dopant-semiconductor complexes. (**a**) Energy-level splitting upon CPX formation schematically illustrated for 4T/F4TCNQ together with DFT-calculated isosurface plots of the bonding and antibonding supramolecular hybrid orbitals (centre), the HOMO of 4T (left) and the LUMO of F4TCNQ (right); 
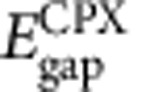
 denotes the transport gap of the CPX and *E*_vac_ the vacuum level. (**b**) UPS/IPES data of 1:1 blends of 4T and F4TCNQ (right) as compared with a pristine 4T reference film (left) demonstrating both an increased IE and a reduced gap upon CPX formation; data are given as binding energy with respect to *E*_vac_; stars mark the experimental onsets of the IPES features.

In preceding work, we have investigated the molecular electrical doping of a number of COMs^29^,^30^ with UV/Vis/NIR signatures similar to the present case of the *p*-doped thiophene oligomer. There, we identified the electronic coupling strength between dopant and COM as a key factor in the fundamental process of molecular doping in oligomeric systems: intermolecular hybridization between the frontier molecular orbitals of COM and dopant leads to a substantial energy-level splitting between a doubly occupied bonding and an empty antibonding supramolecular hybrid orbital in a ground-state charge-transfer complex (CPX)^18^,^29^,^30^,^42^,^43^,^44^,^45^. Instead of ICT with one electron simply hopping from the highest occupied molecular orbital (HOMO) of the COM to the lowest unoccupied molecular orbital (LUMO) of the *p*-dopant, both electrons reside in the bonding hybrid orbital of the CPX, which lies below the HOMO of the circumjacent COM matrix. The empty, antibonding hybrid orbital of the CPX lies, in turn, well above the HOMO of 4T and, therefore, deep within its fundamental gap. This scenario is schematically illustrated in Fig. 4a for F4TCNQ as dopant, together with DFT-calculated isosurface plots of the respective single- and supramolecular hybrid orbitals. On this basis, the sub-gap absorptions in Fig. 3b,d are to be interpreted as transitions between the frontier hybrid orbitals of the CPX, which is corroborated by time-dependent DFT calculations.

Here, to quantitatively determine the individual energies of the CPX states, we carried out ultraviolet (UPS) and inverse (IPES) photoelectron spectroscopy on 1:1 mixed films of 4T and F4TCNQ. Figure 4b compares the results with that obtained for a pristine 4T reference sample. Fully in line with the notion of CPX formation (*cf*. Fig. 4a), our UPS data demonstrate the IE of the 1:1 blend (5.90 eV) to be substantially higher than that of neat 4T (5.30 eV). In combination with IPES, these data further evidence that, in accordance to the UV/Vis/NIR data in Fig. 3b,d, the transport gap of the complex 
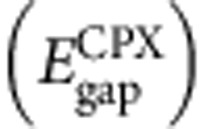
 is substantially narrower than that of neat 4T. For the pristine material, we find an energy separation between the onsets of the HOMO (UPS) and the LUMO (IPES) of 2.70 eV. For the 1:1 blend, however, the separation between the highest/lowest binding energy features in UPS/IPES, assigned to bonding and antibonding supramolecular hybrid orbitals, respectively, is reduced to 0.90 eV; comparison with the onset of optical absorption in Fig. 3a, extrapolated to ∼0.65 eV, thus yields an exciton binding energy of ∼0.25 eV in the CPX, which is small compared with typical COMs^46^,^47^, potentially pointing towards exciton delocalization in the CPX.

Recently, we have shown that 
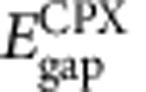
 and its dependence on the dopant EA can be described by a Hückel-like model^29^:


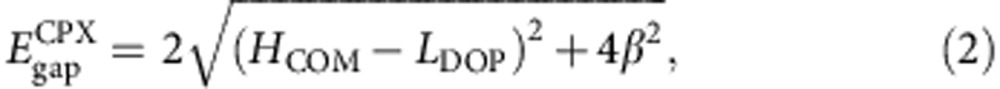


where *H*_COM_ and *L*_DOP_ denote the HOMO energy of the COM and the LUMO energy of the *p*-dopant, respectively. The resonance integral *β*, taking into account the intermolecular electronic coupling between the two, evaluates to 0.42 eV for the CPX of 4T and F4TCNQ. It is noteworthy that, with this constant value for *β* and an equally constant exciton binding energy, the transitions energies of the sub-gap optical absorptions for the entire series of the differently strong dopants (*cf*. Fig. 3) can be remarkably well reproduced (Supplementary Fig. 6).

**Figure 5:**
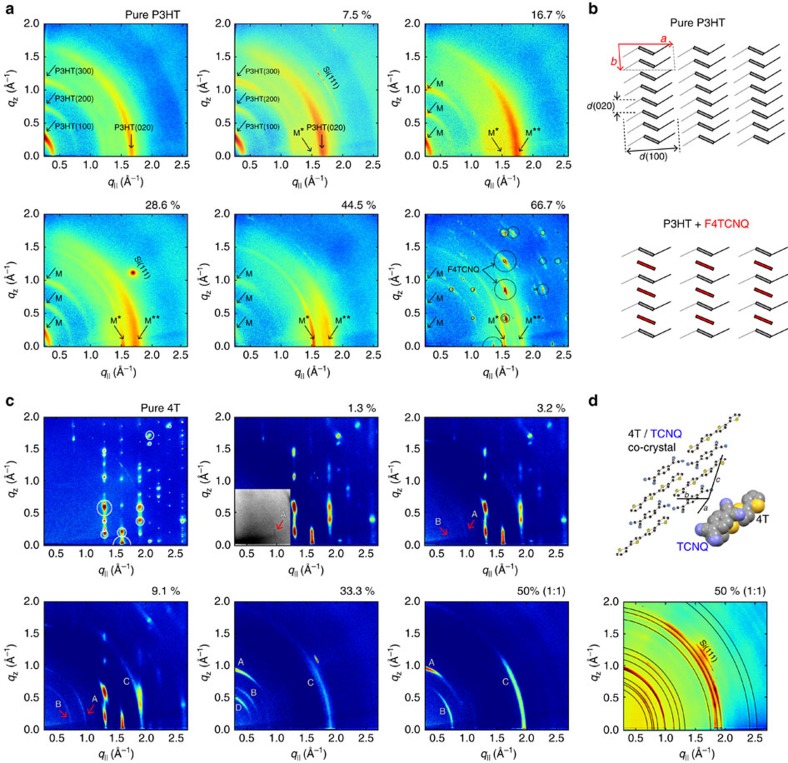
Crystal structure and texture of increasingly doped semiconductor films. (**a**) GIXRD measurements of pure and increasingly doped P3HT films; diffraction features assigned to a mixed phase of P3HT and F4TCNQ are labelled by M, M* and M** (see text). *q*_||_ and *q*_z_ are the in-plane and out-of-plane components of the scattering vector, respectively; areas of the circles correspond to simulated intensities (for precipitated pure F4TCNQ in a (100)-fibre texture)^57^; the dopant ratio is given as number of dopants divided by the sum of dopant molecules and quaterthiophene segments of the polymer chain. (**b**) Schematic model for the solid-state packing of pure P3HT (top)^54^ and in P3HT/F4TCNQ blends (bottom) as viewed along the polymer chain (crystallographic *c*-direction); grey and red boxes represent P3HT and F4TCNQ, respectively, black and grey hexyl chains are located on neighbouring thiophene units of the P3HT backbone. (**c**) GIXRD measurements of pure 4T and co-evaporated 4T/F4TCNQ films of different dopant ratio; the grey area in the 1.3% data is contrast enhanced. (**d**) Illustration of the known 4T/TCNQ crystal structure^59^ of 1:1 mixed crystals and the mutual arrangement of the individual molecules (top). The corresponding calculated reflections are illustrated by black rings in the GIXRD data of a 1:1 co-evaporated 4T/TCNQ reference film (bottom).

Together, optical and photoelectron spectroscopy clearly evidence strong electronic interaction between the thiophene COM and the dopants. To fully understand the implications of this finding for the mechanism underlying the conductivity increase seen in Fig. 1b, we now focus on the local distribution of the dopants within the host for both types of OSCs—an aspect hitherto rarely discussed in literature^16^,^48^. In this context, one might assume (i) the individual dopants to be uniformly dispersed within the semiconductor matrix. This is clearly the case in doped inorganic semiconductors, and, likewise, plausible for OSCs either if amorphous already in their pristine form^8^,^28^ or rendered amorphous upon dopant admixture^30^,^41^. In addition, however, two further microstructural scenarios appear plausible owing to the anisotropic shape of the compounds and the complex mixing behaviour resulting thereof^49^,^50^,^51^,^52^: microphase separation might occur between (ii) pure OSC and dopant domains, and (iii) between pure OSC domains and a stoichiometrically mixed-film portion, as reported in previous studies for both types of OSCs^16^,^29^,^52^. While (ii) clearly disagrees with our FTIR results, where essentially no contribution of the charge-neutral dopants was observed for both OSCs, scenarios (i) and (iii) remain, in principle, compatible with the spectroscopic findings outlined above. To differentiate between the two, we first performed grazing-incidence X-ray diffraction (GIXRD) on F4TCNQ-doped P3HT and 4T films; the results are depicted in Fig. 5.

### X-ray diffraction

For pure P3HT, we observe the diffraction features typical for crystalline, edge-on-oriented P3HT in a (100)-fibre texture (Fig. 5a). The features in *q*_*z*_ direction at low *q*_||_ are assigned to the (*h*00) out-of-plane series with a lattice spacing *d*(100) of 16.2 Å, the characteristic feature at *q*_||_=1.65 Å^−1^ (lattice spacing 3.81 Å) is assigned to the in-plane (020) reflection, that is, the π-stacking distance between neighbouring polymer backbones^53^. All values agree well with numerous reports on pristine P3HT, where a lamellar structure is typically assumed (denoted as a type-I structure with non-interdigitated alkyl chains)^53^,^54^,^55^,^56^ with the thiophene backbones tilted by ca. 26° with respect to the crystallographic *b* axis, as illustrated in Fig. 5b (top)^56^. Upon F4TCNQ admixture, a faint new in-plane feature emerges (labelled as M* in Fig. 5a) with an increased lattice spacing of 4.11 Å, while the (020) reflection of P3HT is significantly broadened, indicating less order in the doped film. This new feature then increases in intensity with dopant loading while retaining its *q*_||_ position for all higher dopant concentrations. The out-of-plane (*h*00) peak series (labelled as M), however, appears at lower *q*_z_ values (for ≥16.7% ratio) and *d*(100) is, therefore, increased to 18.0 Å. Concomitantly, the strong in-plane feature initially assigned to (020) of pristine P3HT is now observed at higher *q*_||_ (labelled as M**) and, finally, can be resolved into two separated diffraction features (≥44.5% ratio), which correspond to 3.74 and 3.53 Å in lattice spacing (Supplementary Fig. 7). Finally, for the highest dopant ratio of two dopants per 4T backbone segment (66.7% ratio), the pattern of pristine, crystalline F4TCNQ in its known crystal structure is observed^57^, which evidences dopant precipitation. We interpret these data as formation of a mixed-crystalline phase of P3HT and F4TCNQ, where the dopant molecules are sandwiched between neighbouring P3HT backbone chains, as schematically illustrated in Fig. 5b (bottom), the precise structure of which, however, is not accessible from our present data. In particular, the finding that all diffraction features assigned to the mixed phase (M, M* and M**) appear independent from the dopant loading, can be understood as F4TCNQ molecules gradually filling the available space in between neighbouring CP backbones until precipitation in the overdoped system occurs. For a ratio of one dopant per 4T backbone unit, the most regular lattice is then expected from this model, which is in line with the well-defined diffraction features in the 44.5% case, where M* then might be assigned to (020) of the mixed-crystal structure and M** to respective mixed-index peaks (0*kl*). We note on the side that these findings qualitatively agree well with a previous report by Duong *et al*.^16^, who, likewise, deduced the formation of a mixed-crystalline phase for this system on the basis of very similar data despite elevated temperature during film preparation. Our data, hence, support the above scenario (iii) with the formation of a stoichiometrically mixed-film portion.

For the pure 4T oligomer, GIXRD yields sharp peaks on vertical rods (Fig. 5b) indicative of standing molecules grown in a (001)-fibre texture of a 4T polymorph reported by Siegrist *et al*.^58^ Upon F4TCNQ admixture in a low ratio of 1.3%, a weak feature (A) appears at low *q*_||_-values (see inset with enhanced contrast in Fig. 5c). It increases in intensity for higher dopant loading and is accompanied by additional reflections (B, C and D), none of which can be explained by any known polymorph of pure 4T or pure F4TCNQ. Concomitantly, all features become smeared out on rings of a constant scattering vector 
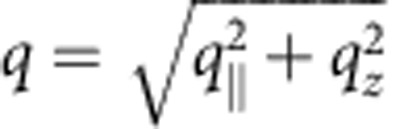
, which indicates a loss of texture. Finally, in the 1:1 case, three dominant features (A, B and C) remain. For the related system 4T/TCNQ, a single-crystal solution for 1:1 mixed crystal exists^59^, which perfectly allows indexing all observed reflections of an identically prepared reference film of 4T/TCNQ; the positions of the calculated peaks are illustrated by black rings (Fig. 5d). Because the GIXRD data of the 1:1 mixed film of 4T/F4TCNQ (Fig. 4a) clearly resembles that pattern, we attribute the features (A, B and C) to essentially isostructural 1:1 mixed crystallites of 4T/F4TCNQ; similar scenarios are found for the entire series of differently strong dopants (Supplementary Fig. 8). In contrast to both pure 4T and all pristine dopants, where neighbouring molecules adopt a herringbone arrangement, this mixed-crystal structure is characterized by a co-planar π-stacking of alternating 4T and dopant molecules, as illustrated in Fig. 5d for 4T/TCNQ. Even more clearly than in the case of P3HT, our GIXRD analysis supports scenario (iii) also for the *p*-doped oligomer with phase separation between pristine 4T and 1:1 mixed crystallites of the CPX already at a concentration as low as 1.3%, which is well within the initial rising edge of the conductivity (*cf*. Fig. 1b).

### Atomic force microscopy

**Figure 6:**
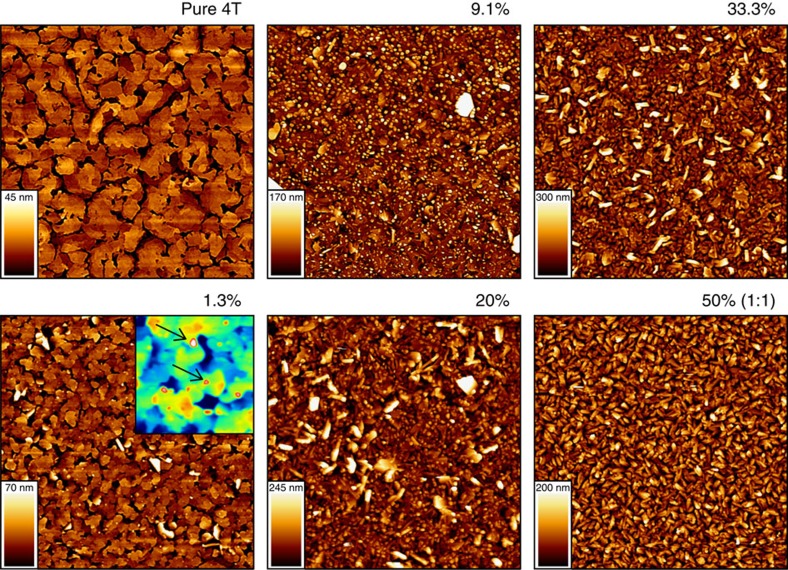
Morphology of increasingly doped 4T films. AFM micrographs (10 × 10 μm) of co-evaporated 4T/F4TCNQ films with increasing dopant ratios; the inset shows a zoom of 2 × 2 μm with enhanced colour contrast and arrows point to features assigned to precipitated 4T/F4TCNQ co-crystals of 1:1 stoichiometry.

As conductivity is (mobile) charge-carrier density times their mobility, the observed increase in *σ* could, in principle, not only be caused by a doping-related increase in carrier density, but also be due to an improved film morphology/structure upon doping, as it was, indeed, suggested for F4TCNQ-doped P3HT^60^ without, however, the occurrence of exceptional features in topographic atomic force microscopy (AFM) images even at high doping levels^48^. To contrast these findings against the oligomer system and to complement our X-ray results, we present AFM results on increasingly F4TCNQ-doped 4T films in Fig. 6. The pure 4T reference shows a morphology with monomolecular steps of 1.5±0.1 nm reminiscent of upright standing molecules, which is in line with the GIXRD data evidencing growth in (001) texture with a lattice spacing of 1.523 nm. Upon dopant admixture, the 4T island size is significantly reduced which, therefore, increases the density of grain boundaries in the doped film. Clearly, this change in morphology must be expected to be detrimental to charge-carrier mobility and, thus, to the conductivity of the film. In line with the GIXRD data pointing towards the presence of the 1:1 mixed co-crystals of 4T and F4TCNQ already at the lowest dopant ratio of 1.3%, a second, granular morphology emerges (inset shows zoomed part with enhanced contrast), which increases in concentration with the dopant ratio. Finally, in the 1:1 situation, only a single morphology is observed, which we now can confidently assign to the 1:1 mixed crystallites. An analogous behaviour was observed for all combinations of 4T with the differently strong dopants (Supplementary Fig. 9). Overall, both the phase separation and the changes in morphology observed here are expected to reduce charge-carrier mobility. Consequently, the increase in conductivity in the F4TCNQ-doped 4T films is due to an over-compensating increase in the density of mobile charges, which must stem from the presence of the 1:1 co-crystallites.

## Discussion

**Figure 7:**
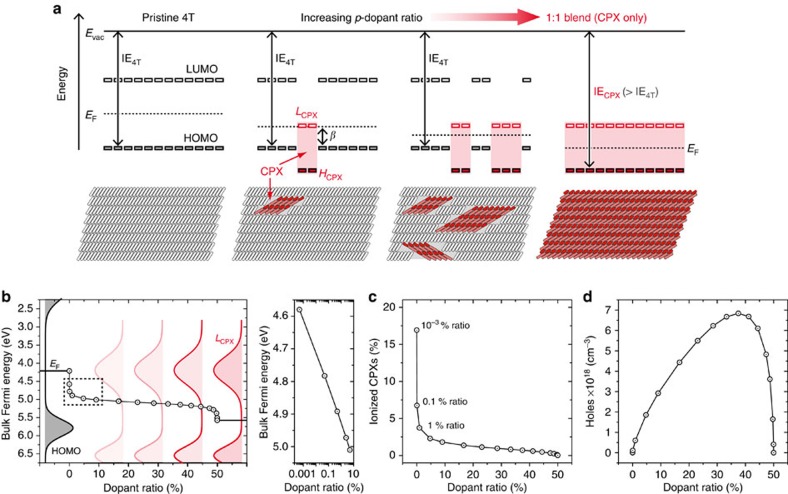
Evolution of electronic structure with increasing doping level. (**a**) Schematic energy level diagram of CPXs embedded into a 4T matrix; the lower panels show the proposed microstructure of the respective systems. (**b**) Left: calculated energy of the bulk Fermi level (*E*_F_) as a function of dopant ratio; grey- and red-shaded areas represent the 4T and 4T:F4TCNQ co-crystal density of states, respectively. The region of low dopant ratios (dashed box) is additionally represented on a logarithmic concentration scale (right). (**c**) Calculated fraction of negatively charged CPXs as a function of dopant ratio. (**d**) Calculated hole density in the 4T matrix as a function of dopant ratio.

In contrast to what is seen in P3HT^16^,^25^,^26^, our results point towards the fact that it is not individual F4TCNQ molecules that should be regarded as the dopants in the oligomer film but, rather, the CPX crystallites. To rationalize the mechanism by which their peculiar electronic structure (*cf*. Fig. 4a) leads to an increase in the density of mobile charge carriers, a qualitative picture—shown in Fig. 7a in its adaption to the present case—has been previously proposed^14^,^29^,^30^,^42^. The unoccupied supramolecular hybrid orbitals of the CPXs (*L*_CPX_) energetically lie by at least the value of the resonance integral *β*≈0.4 eV above the HOMO of the circumjacent 4T film, that is, at least one order of magnitude farther away than typical acceptor levels in *p*-doped inorganic semiconductors lie above their valence band edge. Therefore, only a fraction of the *L*_CPX_ states are occupied at room temperature, leading to negatively charged 1:1 co-crystallites and mobile holes within the 4T matrix. Upon increasing concentration of CPX crystallites, the Fermi level moves down in energy until it is stabilized between the unoccupied hybrid orbitals and the HOMO of the COM.

Here, to quantify the consequences of this picture, both the HOMO- and LUMO-level distributions in the pure 4T and the energy distributions of the occupied/unoccupied hybrid orbitals of the CPX co-crystals were assumed to be reasonably well represented by Gaussian functions^61^,^62^,^63^, whose respective peak centres and standard deviations (s.d.) were extracted from the experimental data in Fig. 4b (see the Methods section). With the molecular volume densities known from the respective single-crystal structures^58^,^59^, an accordingly weighted superposition of the densities of states (DOS) of each individual material was then constructed for a series of dopant ratios. For each dopant ratio, the bulk Fermi level (*E*_F_) and the individual occupations of all four Gaussian energy-level distributions contributing to the mixed DOS were then obtained by repeatedly solving the Gauss–Fermi integrals numerically until the point of overall charge neutrality was found.

The results in Fig. 7b show that, at ultralow dopant ratios of 10^−3^ %, *E*_F_ still lies well within the LUMO distribution of the CPXs (*L*_CPX_), but soon stabilizes between the *L*_CPX_ and the 4T HOMO distributions over a wide range of dopant ratios until it eventually drops further to its bulk value in the pure CPX co-crystal. This evolution entails that, in contrast to typical dopants in inorganic semiconductors, the fraction of charged CPXs is never unity to begin with and rapidly drops with increasing dopant ratio (Fig. 7c). Note that the occupation of states other than the HOMO of 4T and *L*_CPX_ is essentially negligible (Supplementary Fig. 10). Because the number of CPXs increases relative to the number of 4T molecules, the volume density of holes in the doped organic film initially rises and shows a pronounced maximum at a dopant ratio of 38%, around where also the maximum in conductivity was observed in Fig. 1b. Note, however, that the maximum value of ca. 7 × 10^18^ cm^−3^ by no means refers to the density of mobile holes only, but that it includes also those that are likely trapped in the (localized) states that make up the low binding energy tail of the 4T HOMO-level distribution^64^,^65^,^66^. Upon further increasing the dopant ratio, the hole density then rapidly drops again to reach negligible values for the pure 4T/F4TCNQ co-crystal, rationalizing the lower conductivity in this limiting case independent of the potential impact of morphology (*cf*. Fig. 6). We stress that the doping mechanism outlined here for the oligomer is fundamentally different from ICT in the molecularly doped polymer, in that it entails fundamentally different demands for the design of efficient molecular dopants. For the oligomer, minimizing the resonance integral *β* emerges as the key strategy for improving the doping efficiency, because its value limits how closely *L*_CPX_ can approach the occupied states of the COM in energy (Fig. 7a) and, therefore, how many charge carriers are created at room temperature through Fermi–Dirac occupation of all available states (Fig. 7b–d).

To summarize, with the aim of comparing the molecular doping of conjugated oligo- and polymers, we juxtaposed the *p*-doping of P3HT with F4TCNQ, where ICT was reported to occur localized to one 4T unit of the polymer chain, to that of the 4T oligomer itself, which has been regarded as a model system for the polymer. Overall, we observed a vastly different phenomenology for the two materials classes: comparably to P3HT, doping 4T films increases their lateral conductivity by several orders of magnitude despite a severe doping-induced deterioration of their texture and morphology. In contrast to P3HT, however, surprisingly small shifts of the characteristic cyano-vibrational bands in the F4TCNQ dopants reveal that, instead of ICT, only partial charge transfer (*δ*≤0.25) occurs for the 4T oligomer. Moreover, the well-defined spectral features observed in FTIR point towards an equally well-defined mutual orientation of the 4T and dopant molecules. This is corroborated by combining GIXRD with morphological investigations, where we observe phase separation between pure 4T and 1:1 co-crystalline regions of 4T/F4TCNQ already at low doping concentrations. In full analogy, the formation of such a mixed-crystalline phase is also deduced from our GIXRD data of *p*-doped P3HT. In marked contrast to P3HT, however, where the polaron signatures of the individual components dominate, optical absorption spectroscopy evidences strong frontier-orbital hybridization between oligothiophene and dopant in the co-crystallites, which is supported by a theoretical treatment on the DFT level. The resulting supramolecular hybrid energy levels of these ground-state charge-transfer CPXs were directly observed by UPS and IPES. Consequently, instead of the individual dopant molecules being ionized through ICT, as in the case of the polythiophene, it is the co-crystallites of CPXs that are being ionized in the case of the oligomer, thereby effectively acting as dopants by generating holes in the circumjacent COM film. Our study thus highlights profound differences between the fundamental doping mechanisms at work in small molecular and polymeric OSCs of identical chemical composition and similar microstructure. In particular, π-stacking of dopant and quaterthiophene unit entails a substantial resonance integral *β* with the oligomer (with all the consequences described above), while, for the polymer, this is either not the case or does not prevent ICT there. Further exploring and rationalizing these differences constitutes an important thrust area of future research, which must aim at deriving a unified picture of molecular electrical doping valid for all OSCs, that is, molecular and polymeric ones.

## Methods

### Experimental

The source materials 4T, TCNQ, FTCNQ, F2TCNQ and F4TCNQ (purity >98%) were purchased at Tokyo Chemical Industry Co., Ltd (TCI) and were used as received; P3HT (Lisicon SP001) was acquired from Merck KGaA, Darmstadt, Germany. If not stated differently, all (doped) 4T samples were prepared at room temperature via vacuum co-deposition of the individual materials (base pressure <10^−8^ mbar) from resistively heated quartz crucibles; the thickness was measured by a quartz-crystal microbalance; the 4T deposition rate was set to 0.04 nm s^−1^ and the rate of the respective dopant was varied to achieve the desired mixing ratio in the film. Solution-processed samples of (doped) P3HT and 4T (Supplementary Fig. 2) were prepared via spin-coating (10 Hz) from mixed CHCl_3_ solutions of the desired ratio of 4T/P3HT and the respective dopant.

Dopant ratios are related to quaterthiophene units throughout the manuscript. For 4T, a ratio of, for example, 1 dopant per 10 host molecules is given as percentage of dopants per total number of molecules, that is, 1/(10+1)=9.1%. To allow for full comparability of the ratios between oligo- and polymer, the ratio is analogously related to 4T segments of the polymer backbone; that is, one dopant per four thiophene monomer units of the polymer backbone equals a dopant ratio of 50%.

The conductivity data (Fig. 1b) were determined for films between interdigitated indium tin oxide contacts (channel width: 1.0±0.1 cm, channel length: (2.0±0.5) × 10^−2^ cm, active layer thickness: (1.30±0.15) × 10^−5^ cm) using a Keithley SourceMeter 2400; the error of the resistivity values determined therewith was the s.d. of several measured samples (2-4) with each measurement done on 10 parallel resistors located between the interdigitated contacts..

FTIR spectroscopy was performed *in vacuo* using a Bruker IFS-66v spectrometer with a mid-range mercury cadmium telluride detector cooled with liquid nitrogen; samples were prepared on non-doped silicon wafers (Siegert prime grade, 1 mm thickness, native oxide, used as received) via drop-casting.

UV/Vis/NIR was performed in transmission under ambient conditions using a PerkinElmer Lambda 950 spectrometer for films prepared on solution-cleaned (acetone, isopropanol and deionized water (in that order), all in ultrasonic bath) QX-type quartz (Präzisions Glas & Optik GmbH, Germany).

Photoemission spectra (UPS) were collected in normal emission with a SPECS Phoibos 100 hemispherical electron energy analyzer on samples prepared on indium tin oxide substrates (pure 4T: drop-cast from chloroform solution; 1:1 blend: vacuum co-deposited). The secondary electron cutoff (for the determination of the vacuum level) was measured with −10 V bias applied to the sample. IPES was performed using an incident electron energy range of 5–15 eV and a bandpass detector employing a NaCl-coated photocathode as a high-pass filter and the transmission cutoff of a SrF_2_ window as a low-pass filter.

GIXRD experiments on 4T blends with F4TCNQ (Fig. 5a) were performed at Helmholtz Zentrum Berlin für Materialien und Energie GmbH (HZB, BESSY II) beamline KMC-2 using a primary beam wavelength of 0.1 nm and a Vantec 2000 area detector (sample to detector distance: 336 mm); the set-up allowed covering a range in reciprocal space of ca. 2 Å^−1^ both in *q*_||_ and *q*_*z*_ direction in a single experiment. The reciprocal space maps were background corrected with a reference scan on bare SiO_*x*_ of identical integration time (1,500 s). GIXRD experiments on 4T blends with TCNQ (Fig. 5b), FTCNQ, F2TCNQ, F4TCNQ and F6TCNNQ (Supplementary Fig. 8) as well as specular X-ray diffraction measurements were performed at beamline ID10 at the European Synchrotron Radiation Facility using a primary beam energy of 22 keV and a three-module DECTRIS Pilatus 300-K detector; data treatment was done with custom-made software; void areas between the modules were corrected for by repeated exposure (30 s) at different detector positions. All samples for GIXRD were prepared via vacuum co-deposition on SiO_*x*_ substrates (Siegert prime grade, native oxide, used as received). For reducing sample degradation, all experiments in both facilities were performed under inert gas (He/Ar) flux; repeated measurements did not show evidence for significant degradation under the measurement conditions employed.

AFM was done on the samples prepared on SiO_*x*_, as used for GIXRD, employing a NanoWizard 3 AFM (JPK Instruments AG, Berlin, Germany). Silicon cantilevers with a spring constant of 42 N m^−1^ and a resonance frequency of 350 kHz were used in tapping mode; data were treated using the software Gwyddion^67^.

### Theory

Following Zhu *et al*.^45^, DFT calculations were performed on isolated 4T/F4TCNQ dimers in the gas phase. In contrast to their work, however, the positions of all carbon, sulfur and nitrogen atoms were extracted from the single-crystal solution of the isostructural 4T/TCNQ case^59^. The missing hydrogen (on the 4T) and fluorine atoms (F4TNCQ) were then added ‘by hand' and fully relaxed to their equilibrium positions. For this geometry optimization, the van der Waals and long-range corrected ωB97X-D exchange-correlation functional^68^ was employed in conjunction with a 6–31G** basis set. Subsequent ground- and excited-state properties (the latter with time-dependent DFT) were performed with the PBE0 functional^69^. The ground-state charge transfer of 0.24 between 4T and F4TCNQ was determined by averaging the results of the Mulliken (0.23), Löwdin (0.24), Hirshfeld (0.22), electrostatic potential fitting (0.26) and natural population analysis (0.24) partitioning schemes as implemented in Gaussian 09, Rev. A02 (ref. 70).

The parameters for the Gaussian DOS, needed to compute the numerical results presented in Fig. 7, were extracted from the data in Fig. 4b. For both pure 4T (UPS) and the pure CPX film (UPS and IPES), the respective frontier-orbital peaks were fitted with a Gaussian yielding the respective peak centres and standard variations *σ*_sum_. As peak-centre positions, we determined 5.79 eV (4T) and 6.54 eV (CPX) for the HOMO levels as well as 4.2 eV (CPX) for the LUMO. Independently, via UPS/IPES on a (polycrystalline) gold reference sample, we determined the instrumental broadening of our set-up by the numeric convolution of a Fermi function with a Gaussian yielding a s.d. of *σ*_exp_ (UPS: 73 meV, IPES: 390 meV). This then finally allowed determining the s.d. *σ*_int_ due to the intrinsic energetic disorder in the film via: 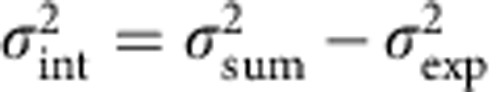, which then was used as the s.d. of the Gaussian functions in our numerical modelling (4T: 0.246 eV (UPS) and 0.375 eV (IPES); CPX: 0.258 eV (UPS) and 0.375 eV (IPES)); note that, as no clear features were observed in IPES for pure 4T, *σ*_int_ determined for the CPX was also taken for the LUMO of 4T. The centre of the 4T LUMO distribution was assumed at 1.85 eV, that is, at a distance 2*σ*_sum_ off the experimentally determined onset (marked with a star in Fig. 4b). For the impact of varying *σ*_int_ and *β* on the calculated hole density in the 4T host, see Supplementary Fig. 11.

## Additional information

**How to cite this article:** Méndez, H. *et al*. Charge-transfer crystallites as molecular electrical dopants. *Nat. Commun*. 6:8560 doi: 10.1038/ncomms9560 (2015).

## Supplementary Material

Supplementary Information Supplementary Figures 1-11, Supplementary Table 1 and Supplementary References
