# Supplementary material for: Charge-transfer crystallites as molecular electrical dopants
Source: Nat Commun. 2015 Oct 6;6:8560. doi: 10.1038/ncomms9560 (PMC4600739; doi:10.1038/ncomms9560)
Supplement: Supplementary Information — Supplementary Figures 1-11, Supplementary Table 1 and Supplementary References [file ncomms9560-s1.pdf]

Supplementary Figures

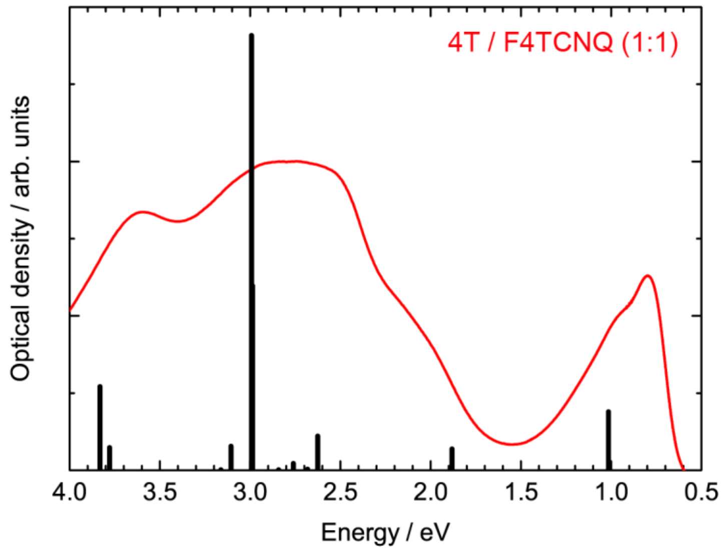

Supplementary Figure 1 | Measured *versus* calculated optical transitions in the CPX. The UV/Vis/NIR spectrum obtained experimentally for the 1:1 blend of 4T and F4TCNQ (red curve) is well reproduced by our time-dependent DFT calculations (for details see the Methods section of the main text). The computed transition energies are indicated by black vertical lines and their heights reflect the corresponding oscillator strengths. That only a single electronic transition is calculated to lie in the range of the lowest-energy CPX absorption allows assigning the fine structure of the experimental data to a vibronic progression.

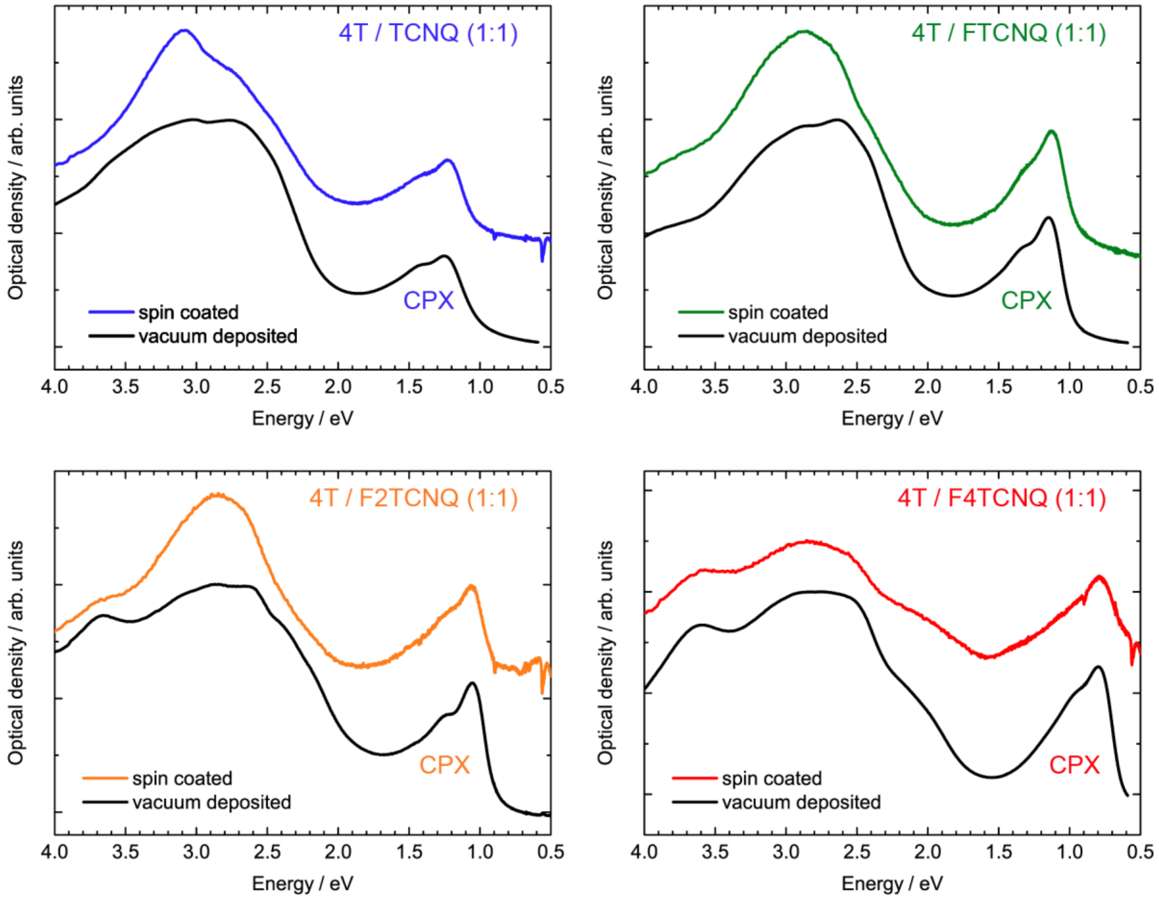

Supplementary Figure 2 | Comparison between UV/Vis/NIR data of solution-processed and vacuum co-deposited films. CPX formation is independent of the preparation technique (spin-coating and vacuum co-deposition), as analogous sub-gap absorptions arise for the entire series of the differently strong dopants TCNQ, FTCNQ, F2TCNQ, and F4TCNQ. The low-energy onsets of the lowest-energy transition are listed in Supplementary Table 1 for all systems.

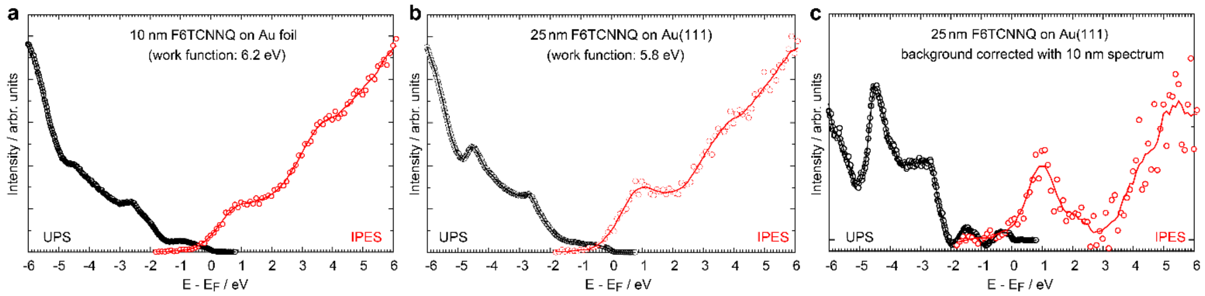

Supplementary Figure 3 | Electronic structure of pristine F6TCNNQ. Combined UPS and IPES data on vacuum deposited F6TCNNQ films on Au yield the *EA* of pristine F6TCNQ. All films [(a): 10 nm, (b): 25 nm nominal thickness] show pronounced island growth, as deduced from the Fermi edge of the substrate being still visible in UPS on the nominally multilayer thick films. The data depicted in (c) are calculated spectra from (b) via background correction with an accordingly shifted and scaled spectrum of (a), which finally yields an *EA* for F6TCNNQ of (5.6 ± 0.2) eV.

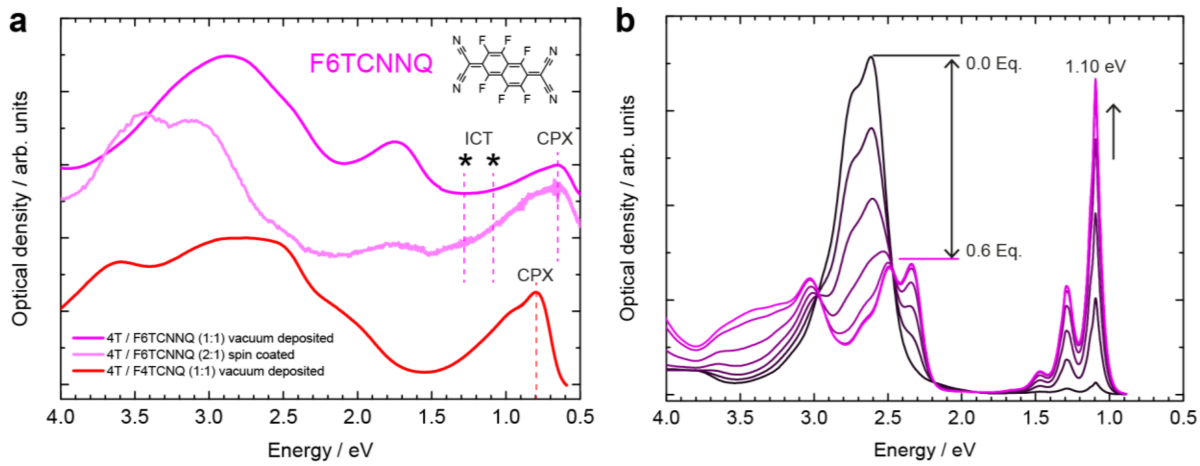

Supplementary Figure 4 | UV/Vis/NIR spectra of 4T/F6TCNNQ blends and F6TCNNQ anions. (a) In full analogy to the TCNQ-series (main text), UV/Vis/NIR spectra of spin coated and vacuum co-deposited blends of 4T with the even stronger electron acceptor 2,2’-(perfluoro-naphthalene-2,6-diylidene)dimalononitrile (F6TCNNQ)[1,2] with an *EA* of 5.6 eV (*cf*. Supplementary Figure 3) shows a new sub-gap absorption that is assigned to the spectral signature of the CPX. The spectrum of 4T/F4TCNQ, where the CPX transitions lies higher in energy, is also shown for comparison (red curve). (b) Evolution of F6TCNNQ absorption spectra upon titration with increasing equivalents (*Eq*) of KI in CH_3CN solution. The lowest energy transition of the radical anions is observed at 1.10 eV; for analogous data on the whole TCNQ series see the Supporting Information of Ref. [2].

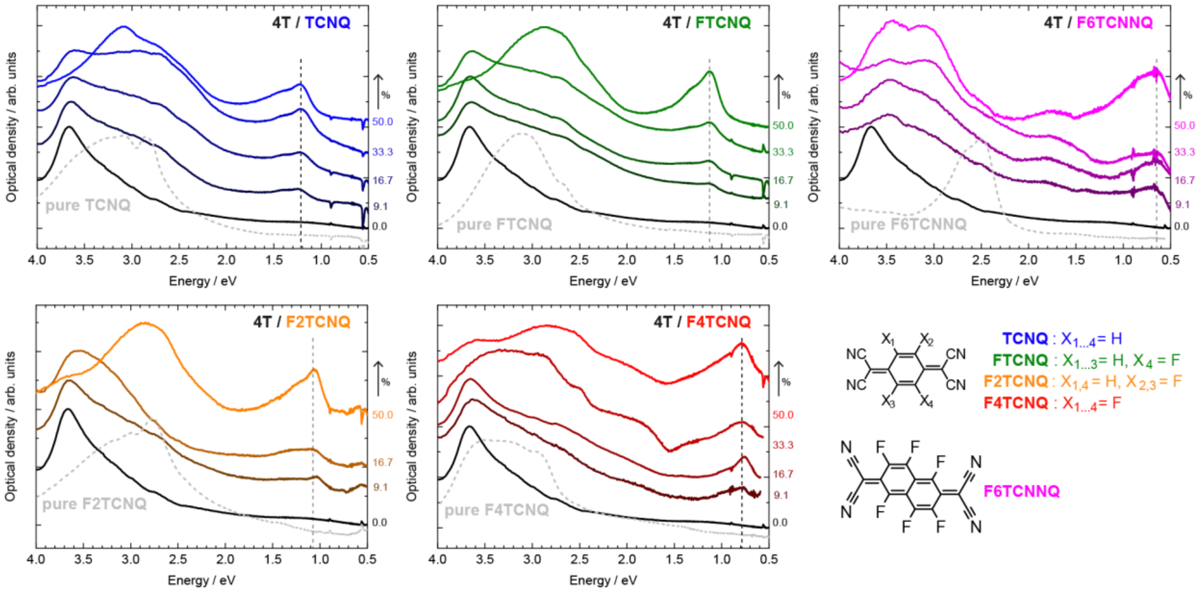

Supplementary Figure 5 | UV/Vis/NIR spectra of increasingly doped (spin-coated) 4T films. The energies of the new sub-gap absorptions assigned to the CPX are marked with vertical lines.

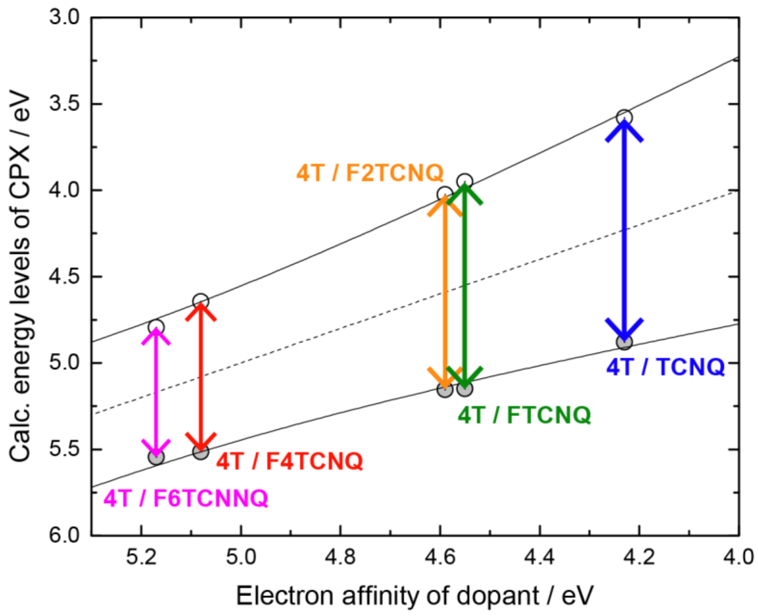

Supplementary Figure 6 | Calculated energy levels of the CPXs. The energy levels of the CPXs formed between 4T and all employed dopants, as calculated by the Hückel-like model (*cf.* equation 2 in the main text), are illustrated as filled (occupied states E_1^{CPX}) and open circles (unoccupied states E_2^{CPX}). The continuous solid lines represent these energies as a function of the dopant EA (i.e. L_{DOP}) according to: $E_{1/2}^{CPX} = \frac{H_{COM}+L_{DOP}}{2} \pm \sqrt{(H_{COM} - L_{DOP})^2 + 4\beta^2}$, where H_{COM} and L_{DOP} denote the HOMO energy of 4T and the LUMO energy of the *p*-dopant, respectively. The resonance integral β was held constant at 0.42 eV, *i.e.*, at the value deduced from the experimental data of 4T/F4TCNQ (main text). This value for β allows well reproducing the energies for the entire dopant series, if an equal exciton binding energy of 0.25 eV is assumed in all cases, which was deduced from comparing the UV/Vis/NIR and the UPS/IPES data of 4T/F4TCNQ.

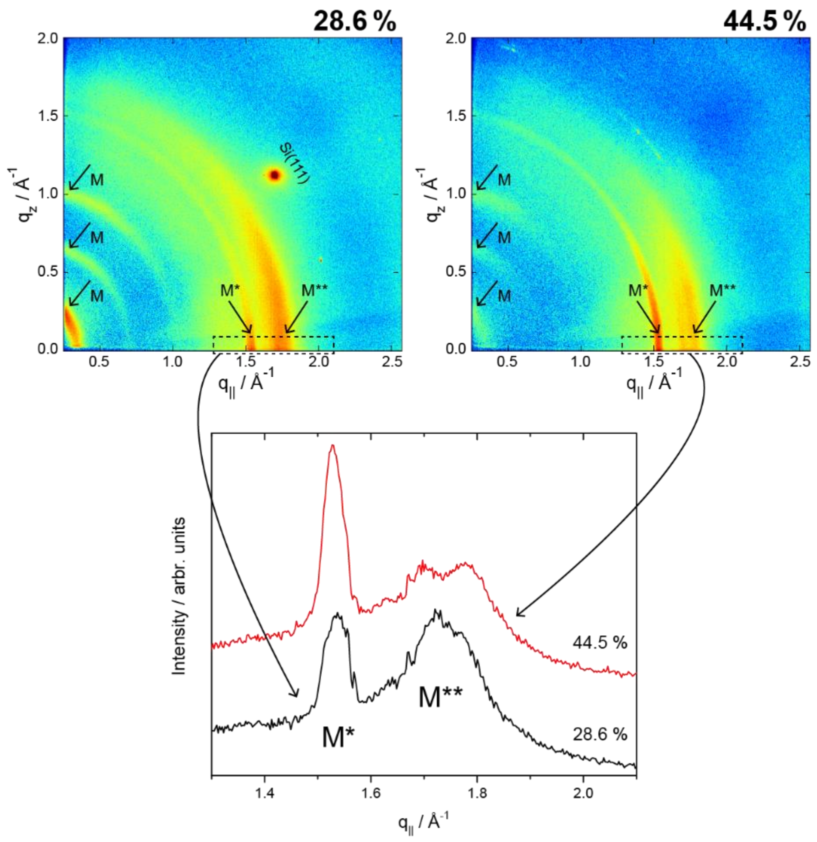

Supplementary Figure 7 | Integrated line-scans of GIXRD data. Integrated line-scans of the 2D GIXRD data of F4TCNQ doped P3HT films (integration along q_z from -0.05 Å^{-1} to +0.05 Å^{-1} in the dashed area of the data) illustrating M** to consist of two components at the high dopant ratio of 44.5%.

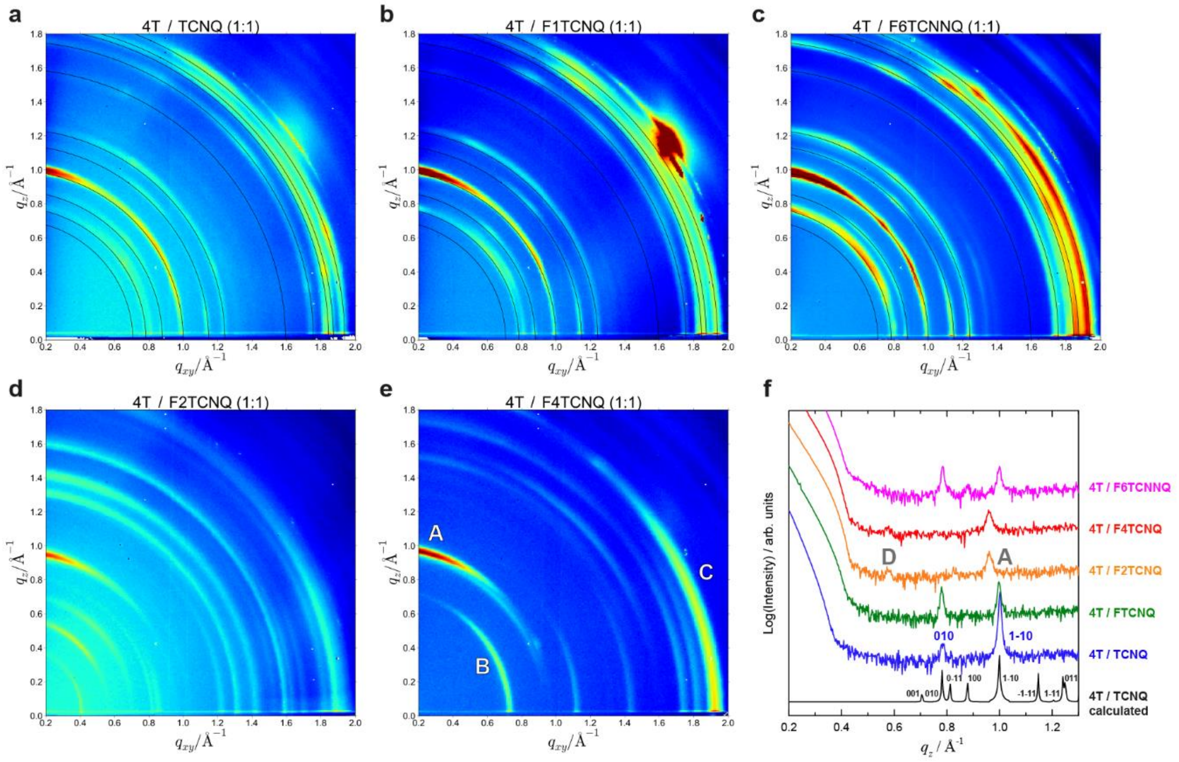

Supplementary Figure 8 | GIXRD and specular XRD data for drop-cast 1:1 blends of 4T with the entire dopant series. (a-e) GIXRD experiments on 4T blends with the whole series of differently strong dopants evidence growth in 1:1 co-crystals of similar structure. From the almost identical patterns (a-c), the 4T/FTCNQ and 4T/F6TCNNQ co-crystals are found to be essentially isostructural to those of 4T/TCNQ, which has been solved by single-crystal X-ray diffraction before (CSD code: FEPYUU);[3] the strongest reflections calculated for this structure are indicated by black rings. In analogy, the most intense features (labeled as A, B, and C in Figure 5a of the main text) are also observed in the patterns of 4T/F2TCNQ and 4T/F4TCNQ (d, e) at similar positions, which points towards growth in similar crystal structures. (f) Specular X-ray diffraction data of all 1:1 films as compared to a simulated powder pattern of the known structure of 4T/TCNQ (black curve). For 4T/TCNQ, 4T/FTCNQ, and 4T/F6TCNNQ, the (010) and (1-10) reflections dominate the spectrum, which indicates (some) preferential orientation in the films. Labels A and D correspond to features also observed in the GIXRD data of 4T/F4TCNQ (Figure 5a of the main text).

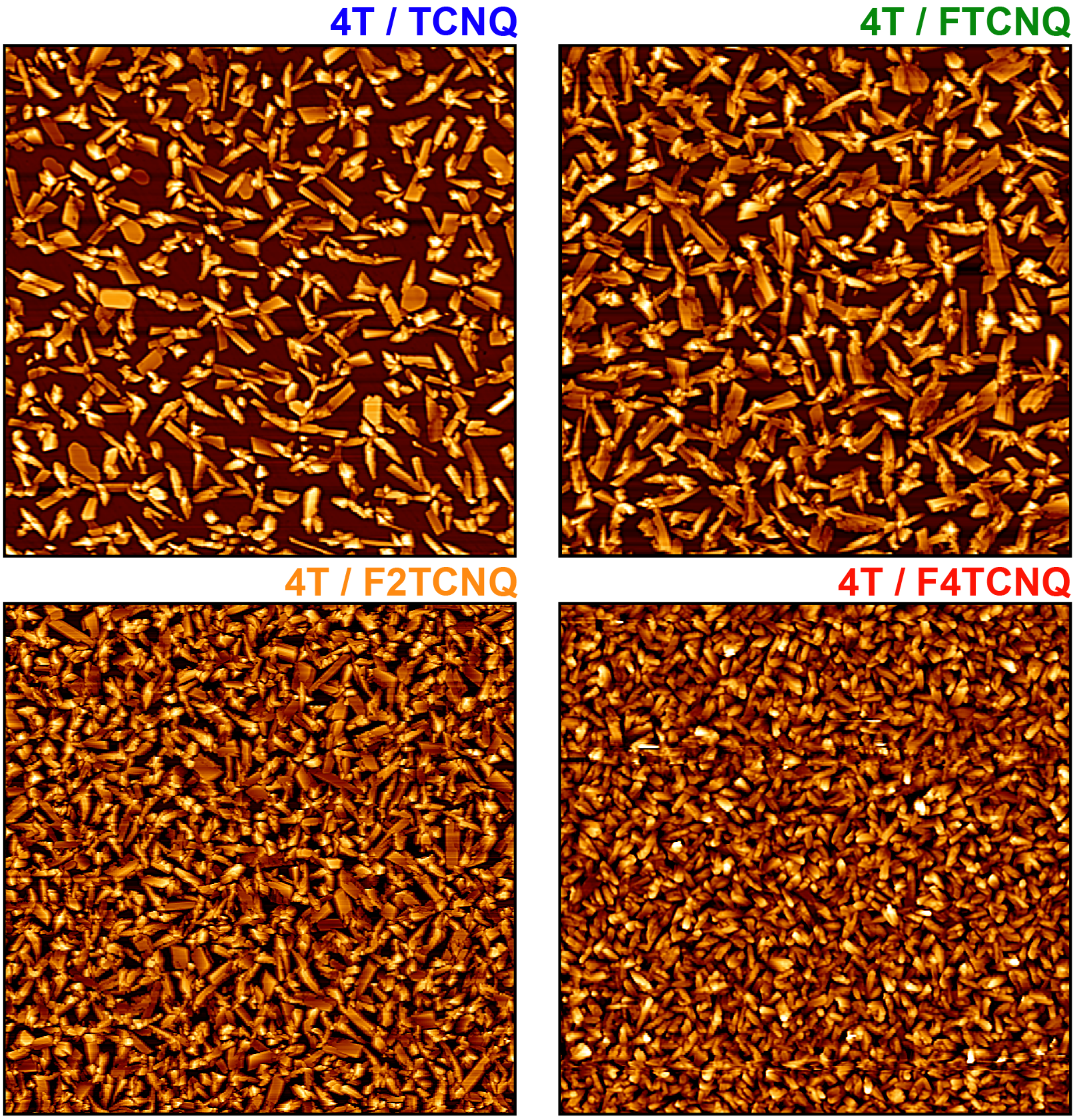

Supplementary Figure 9 | AFM data for 4T doped with the full TCNQ series. AFM micrographs (10 µm x 10 µm) of 1:1 co-evaporated 4T/TCNQ, 4T/FTCNQ, 4T/F2TCNQ, and 4T/F4TCNQ films on SiO_2 substrates. In all cases, only a single morphology is observed, which is assigned to the 1:1 mixed crystallites observed in GIXRD.

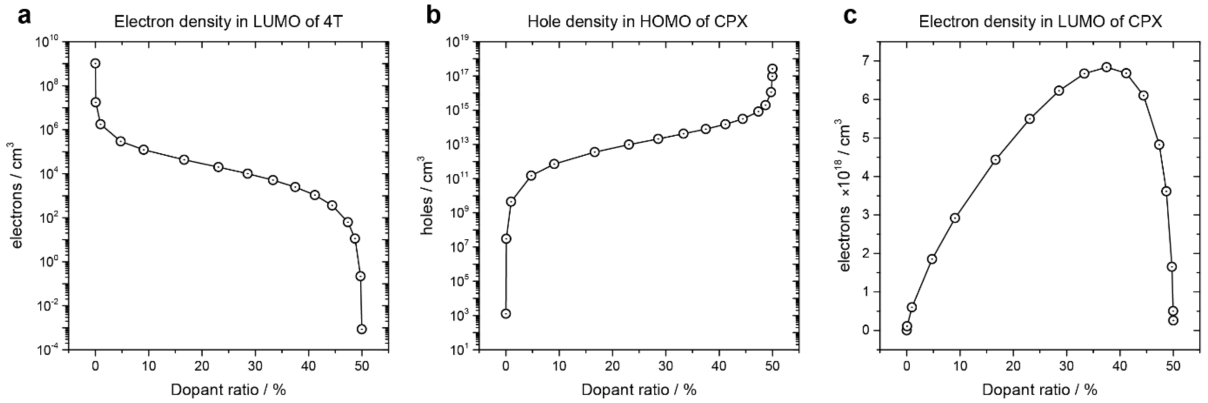

Supplementary Figure 10 | Calculated charge densities in frontier molecular orbitals. (a) Electron density (electrons/cm^3) in the LUMO of 4T. (b) Hole density (holes/cm^3) in the HOMO of the CPX. (c) Electron density (electrons × 10^{18}/cm^3) in the LUMO of the CPX (*cf.* Figure 7d of the main text); data are represented on logarithmic scale for better visibility.

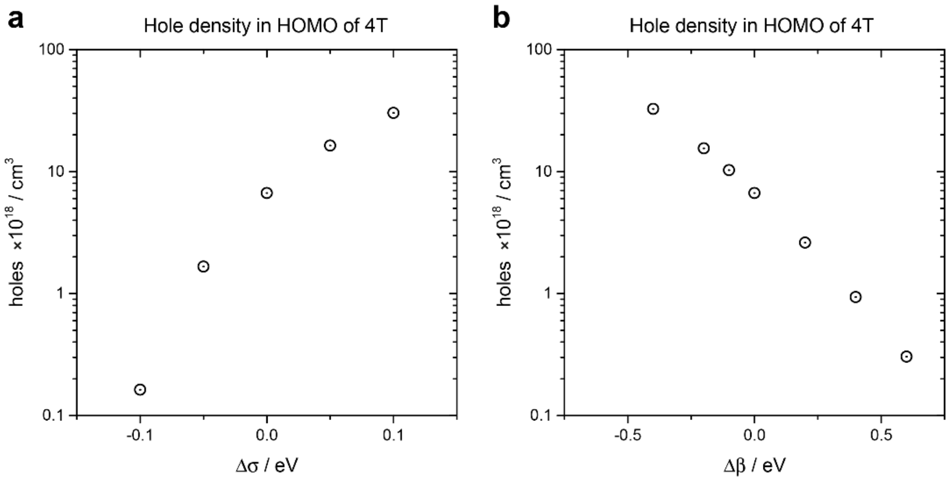

Supplementary Figure 11 | Calculated hole densities in the 4T matrix upon parameter variation. Calculated hole densities (holes × $10^{18}/cm^3$) in the HOMO of 4T under a variation of (a) the standard deviation of all four Gaussian functions by $\Delta\sigma$ and (b), under a variation of the resonance integral β by $\Delta\beta$, i.e., upon varying the position of the CPX LUMO; all calculations were done for a 1:1 mixture of 4T and CPX (dopant concentration of 33.3%).

Supplementary Table

	**4T/TCNQ**	**4T/FTCNQ**	**4T/F2TCNQ**	**4T/F4TCNQ**	**4T/F6TCNNQ**
**vac.**	1.05 eV	0.95 eV	0.88 eV	0.62 eV	0.50 eV
**sol.**	1.02 eV	0.94 eV	0.89 eV	0.60 eV	0.46 eV

Supplementary Table 1 | Transition energies (onsets) extracted from UV/Vis/NIR data of 1:1 blends for vacuum co-deposited (vac.) and solution-processed (sol.) samples.

Supplementary References

1 Kleemann, H. *et al.* Structural phase transition in pentacene caused by molecular doping and its effect on charge carrier mobility. *Org. Electron.* **13**, 58-65 (2012).

2 Méndez, H. *et al.* Doping of Organic Semiconductors: Impact of Dopant Strength and Electronic Coupling. *Angew. Chem., Int. Ed.* **52**, 7751-7755 (2013).

3 Minxie, Q., Heng, F. & Yong, C. The Crystal Structure of Tetrathiophene TCNQ complex. *Chin. J. Struct. Chem.* **5**, 163 (1986).
